# Chronic Hypoxia Impairs Muscle Function in the *Drosophila* Model of Duchenne's Muscular Dystrophy (DMD)

**DOI:** 10.1371/journal.pone.0013450

**Published:** 2010-10-20

**Authors:** Matias Mosqueira, Gabriel Willmann, Hannele Ruohola-Baker, Tejvir S. Khurana

**Affiliations:** 1 Department of Physiology and Pennsylvania Muscle Institute, University of Pennsylvania School of Medicine, Philadelphia, Pennsylvania, United States of America; 2 Department of Biochemistry and Institute for Stem Cell and Regenerative Medicine, University of Washington, Seattle, Washington, United States of America; University of Las Palmas de Gran Canaria, Spain

## Abstract

Duchenne's muscular dystrophy (DMD) is a severe progressive myopathy caused by mutations in the DMD gene leading to a deficiency of the dystrophin protein. Due to ongoing muscle necrosis in respiratory muscles late-stage DMD is associated with respiratory insufficiency and chronic hypoxia (CH). To understand the effects of CH on dystrophin-deficient muscle *in vivo*, we exposed the *Drosophila* model for DMD (*dmDys*) to CH during a 16-day ascent to the summit of Mount Denali/McKinley (6194 meters above sea level). Additionally, *dmDys* and wild type (WT) flies were also exposed to CH in laboratory simulations of high altitude hypoxia. Expression profiling was performed using Affymetrix GeneChips® and validated using qPCR. Hypoxic *dmDys* differentially expressed 1281 genes, whereas the hypoxic WT flies differentially expressed 56 genes. Interestingly, a number of genes (e.g. heat shock proteins) were discordantly regulated in response to CH between *dmDys* and WT. We tested the possibility that the disparate molecular responses of dystrophin-deficient tissues to CH could adversely affect muscle by performing functional assays *in vivo*. Normoxic and CH WT and *dmDys* flies were challenged with acute hypoxia and time-to-recover determined as well as subjected to climbing tests. Impaired performance was noted for CH-*dmDys* compared to normoxic *dmDys* or WT flies (rank order: Normoxic-WT ≈ CH-WT> Normoxic-*dmDys*> CH-*dmDys*). These data suggest that dystrophin-deficiency is associated with a disparate, pathological hypoxic stress response(s) and is more sensitive to hypoxia induced muscle dysfunction *in vivo*. We hypothesize that targeting/correcting the disparate molecular response(s) to hypoxia may offer a novel therapeutic strategy in DMD.

## Introduction

Mutations in the *DMD* gene cause Duchenne muscular dystrophy (DMD), which is associated with a loss or severe reduction of the dystrophin protein [1], [2], [3]. The disease is characterized by severe progressive muscle degeneration. The main causes of morbidity and mortality in DMD patients are cardiac and respiratory muscle failure, the latter estimated to be responsible for ca. 55 to 90% of the cases [4], [5], [6], [7]. Kyphoscoliotic deformities of the spinal column and chest wall [8], [9], [10], reduced mobility along with ongoing necrosis in the respiratory muscles contribute to the reduced vital capacity [11], [12], hypoxemia and hypercapnia [8] noted in DMD patients with respiratory insufficiency. While extremely important from a patho-physiological viewpoint, the possible contribution of chronic hypoxia (CH) to disease progression in DMD patients is not fully understood.

Full length dystrophin is a 427 KDa cytoskeleton associated protein composed of four distinct structural domains: 1) N-terminal “actin binding” domain; 2) rod domain consisting of spectrin-like repeats; 3) cysteine-rich domain; and 4) a carboxyl-terminal domain [3], [13], [14]. Dystrophin is associated with a complex of proteins including the dystroglycans, sarcoglycans, sarcospan, syntrophins, and dystrobrevins to form the called dystrophin associated complex (DAC) [13], [15]. Apart from a structural cytoskeletal role, the DAC has been proposed to have an intracellular signaling role based on its association with acetylcholine receptors [16], [17], voltage-gated sodium channels [18], [19] and the neuronal isoform of the nitric oxide isoform (nNOS) [13], [15], [20], [21], [22]. Muscles from mice deficient nNOS, either by a primary nNOS-specific mutation or secondarily by dystrophin-deficiency, show insufficient production of NO, resulting in impaired metabolic modulation of α-adrenergic vasoconstriction [23], [24], [25]. Consequently, the normally protective vascular relaxation mechanism is compromised and ischemia has been noted in dystrophin-deficient limbs. Indeed, changes in vascular function have been suggested as early and important pathological changes in DMD muscles [26], [27]. Distinct from the localized hypoxia due to inadequate blood supply noted in ischemia (e.g. due to inadequate NOS activity) DMD patients have a well-described ventilatory insufficiency associated with significant desaturation of arterial oxygen (from 95% to 74% during sleep time) and ensuing systemic hypoxia that would also be predicted to contribute to patho-physiology [28], [29]. These issues are also being addressed in various DMD animal models. Indeed, the *mdx* mouse model has alterations in ventilatory frequency and responses to hypoxia compared to normal mouse. Additionally exposure to episodic hypoxia has been noted to exacerbate diaphragmatic dysfunction in *mdx* mice [30], [31], [32].

The dystrophin gene is evolutionarily well conserved and a number of animal models of DMD have been described including mouse (*mdx*) [33], [34], dog (GRMD) [35], zebra fish [36], *C. elegans* [37] and *Drosophila melanogaster* (*dmDys*) [15], [38], [39], [40], [41], [42] with mutations existing in the dystrophin gene in vertebrates or invertebrates genes [15], [43]. The *Drosophila melanogaster* dystrophin homolog shares 54% sequence identity with human dystrophin, having conserved motifs in regions that interact with dystroglycan, syntrophins and dystrobrevins [15]. The organization of the Drosophila dystrophin gene as is complex as its mammalian counterparts, having three large isoforms called dystrophin-like products and three truncated products (Dp186, Dp205 and Dp117) sharing with the large isoforms the cysteine-rich domains and carboxy-termini driven by separate internal promoters [15], [38]. Two isoforms of syntrophin, a protein that interacts with voltage-gated sodium channel and nNOS via PDZ domains, is also expressed in *Drosophila melanogaster* with a 40% identity with human counterparts [15]. NOS is another protein constituent complex of the DAC that is expressed in *Drosophila melanogaster*. Flies have only one single gene for NOS, instead of three found in vertebrates. Flies heterozygous for the NOS deletion show a reduced level of protein and half the wild type level of NOS activity, while homozygous deletion in this gene causes lethality in the offspring [44].

Hypoxia is a potent stimulus known to induce changes in expression of a broad array of genes. Particularly well studied in mammals are cascades of gene expression occurring via activation of the Hypoxia Inducible Factor (HIF) transcription factor pathway. *Drosophila* responds to hypoxia using the *sima* and *tango* pathways which are similar to HIF-1α and HIF-1β, respectively [45]. The Sima response to hypoxia has been characterized using a HRE-LacZ reporter gene for murine lactate dehydrogenase A (LDA) in transgenic fly embryos. Sima expression increased LDA after exposing the transgenic flies to 5% O_2_ for 8 h [46]. The deletion of the oxygen-dependent degradation domain (conserved in mammals and *Drosophila*) induced an increase in Sima protein levels, similar to that seen under hypoxic conditions, demonstrating that Sima is oxygen-dependent and regulated by a mechanism similar to the one described for mammalian HIF-α. The *tango* gene product has structural similarities to its mammalian ortholog HIF-1β protein and is known to binds Sima and other bHLH proteins [47]. Challenging adult *Drosophila* to two different lengths of exposure to hypoxia induces the expression of different sets of genes [48]. Interestingly, in *Drosophila* the heat shock proteins (HSPs) expression was not induced in the mild hypoxia conditions (5% O_2_), but was induced during extreme hypoxic conditions (1% O_2_), suggesting that these gene expression changes are an adaptive response to low oxygen levels rather than a response to changes in exposure time [48].

*Drosophila*, as other insects, depend on pairs of segmental or intersegmental muscles located in the dorsal region of each abdominal segment for several movements, such as ventilation, oviposition and steering during flight and on direct and indirect thoracic flight muscles for flight [49], [50]. Those muscles show age-depended degeneration in dystrophic flies and significantly reduce mobility of the flies [40], suggesting that dystrophic flies may be subject to poor functional recovery from hypoxic challenges if the muscles function were compromised by CH. However, the effects of CH on changes of gene expression and muscle function in dystrophin-deficient states have not been fully characterized. Here, we examine the effects of CH in the *Drosophila* DMD model (*dmDys*), using a combination of molecular and functional assays including Affymetrix GeneChip®-based expression profiling, qPCR, recovery time after an acute hypoxia challenge and climbing assays. Our data demonstrate that dystrophin–deficient flies mount a disparate, pathological response to hypoxia and that chronic exposure to hypoxia impairs muscle function dystrophin-deficient flies *in vivo*.

## Materials and Methods

### *Drosophila* stocks

*UASdmDysC-term*, UAS*dmDysN-term*, *ω^−^;;24B-Gal4* and *P-tub-Gal4* stocks flies were have been previously described [40] and were maintained at room temperature on standard molasses media. For expression profiling and functional experiments, we used dystrophic flies which were obtained by crossing *UASdmDysC-term* with *P-tub-Gal4* (ubiquitous expression of dsRNA to all tissues and hence blocks the expression of all short and long dystrophin isoforms). Additionally, we also used the offspring of *UASdmDysC-term* crossed with the driver *24B-Gal4* (mesoderm muscle expression of dsRNA) and *UASdmDysN-term* (expression of dsRNA to block the expression of all short and long dystrophin isoforms) driven by both drivers for functional experiments. *P-tub-Gal4* driver flies were used as gene expression controls. *P-tub-Gal4* and *24B-Gal4* drivers were used as controls for functional assays, as appropriate.

### Exposure to Chronic Hypoxia

Flies were exposed to CH during a 16-day ascent of Mount Denali/McKinley (6194 meters above sea level) with permission from the National Park Service, USA. All flies were transported by aeroplane from Philadelphia in thermally insulated vials carried on person to Kahiltna Glacier at the base of Mount Denali/McKinley and carried on foot thereafter. Environmental oxygen pressure (PO_2_) was calculated from expedition logs (Table S1) using the formula developed by Prof. John B. West [51]. *DmDys* and WT flies were also exposed to CH in laboratory simulations using PO_2_ values from Table S1 using a Pegas 4000F gas mixer (Columbus Instruments, OH, USA). Normoxic flies were kept breathing at room air in Philadelphia, PA. After exposure to CH, flies were immediately frozen in RNALater® (Ambion, Applied Biosystems, Foster City, CA) and kept frozen till they were processed for gene expression profiling.

### Expression profiling and qPCR validation

Methods were applied as described by manufacturer and carried out in the laboratory and at the University of Pennsylvania Microarray Core. The whole body of each fly was individually homogenized and total RNA was extracted from individual flies using a RNeasyMini kit (Qiagen, Valencia, CA) and 100 ng were amplified using NuGen Ovation Amplification Kit (NuGen, San Carlos, CA). The processed cDNA prepared from 3.75 µg of aRNA was hybridized onto GeneChip® Drosophila Genome 2.0 array platform (Affymetrix, Santa Clara, CA). Microarray images were acquired by GSC 3000 laser scanner (Affymetrix). The raw intensity values were processed and normalized by CG-RMA algorithm from raw *CEL* files and analyzed using GeneSpring GX V 7.3.1 software (Aligent Technologies, Santa Clara, CA). The statistical significance (p<0.05), the false discovery rate-FDR (0%), and the ratio of the changes in expression (2 fold-change cut-off) was calculated using Significance Analysis of Microarray software [52]. Microarray results were validated through real time qPCR; 100 ng of total RNA from four individual flies were used to performed reverse transcriptional reaction using SuperScript First-Strand, random hexamers protocol (Invitrogen, Carlsbad, CA). TaqMan® Gene Expression assays using MGB probe and primers (Applied Biosystems, Foster City, CA) for different genes were performed in a 20 µL final volume (Table S2). The target gene expressions were normalized using ribosomal protein 11 expression. All primary microarrays for the *dmDys* and WT studies describe above is MIAME compliant and the raw data has been deposited at the MIAME compliant NCBI's Gene Expression Omnibus (GEO, http://www.ncbi.nlm.nih.gov/geo/) and are accessible through GEO Series accession number GSE15879.

### Functional assays

The recovery from severe hypoxia assay was performed as previously described [53]. Briefly, 100 adult (3–5 days-old) flies/genotype were placed in 5 different tubes (20 flies/tube) covered by air permeable cloth and exposed for 2 hours to 1% of FiO_2_ and then returned to room air. The time of recovery was recorded once the fly was able to start to climb up the vial. Each fly was exposed once to the severe hypoxia assay. The climbing assay was performed to measure the upward mobility as previously described [40]. Briefly, five vials from each genotype containing 20 flies each (100 flies in total) were put into a glass 50 mL tube (12 cm long) and tapped once to keep them at the bottom. After 30 seconds, the first tube was blocked with cotton and then the second tube, which was connected to the first tube, was connected to the third tube for another 30 second, and so on. The number of flies in each tube was counted and the climbing index was calculated as weighted average according to the tube number; the sum of the number of flies in each tube multiplied by the tube number and divided by the number of the last tube times the number of the flies in the assay [54], [55]. Each fly was exposed once to the climbing apparatus.

### Statistical analysis

All results were expressed as mean ± SEM. The qPCR results were analyzed using Relative Expression Software Tool-Multiple Condition Solves version 2 (REST-MCS) [56]. Kaplan-Meier test was performed on recovery assay and Kruskall-Wallis test and Dunn's multiple comparisons post-test for climbing assays. A p-value <0.05 was considered statistically significant.

## Results

### Gene expression profile

**Figure 1:**
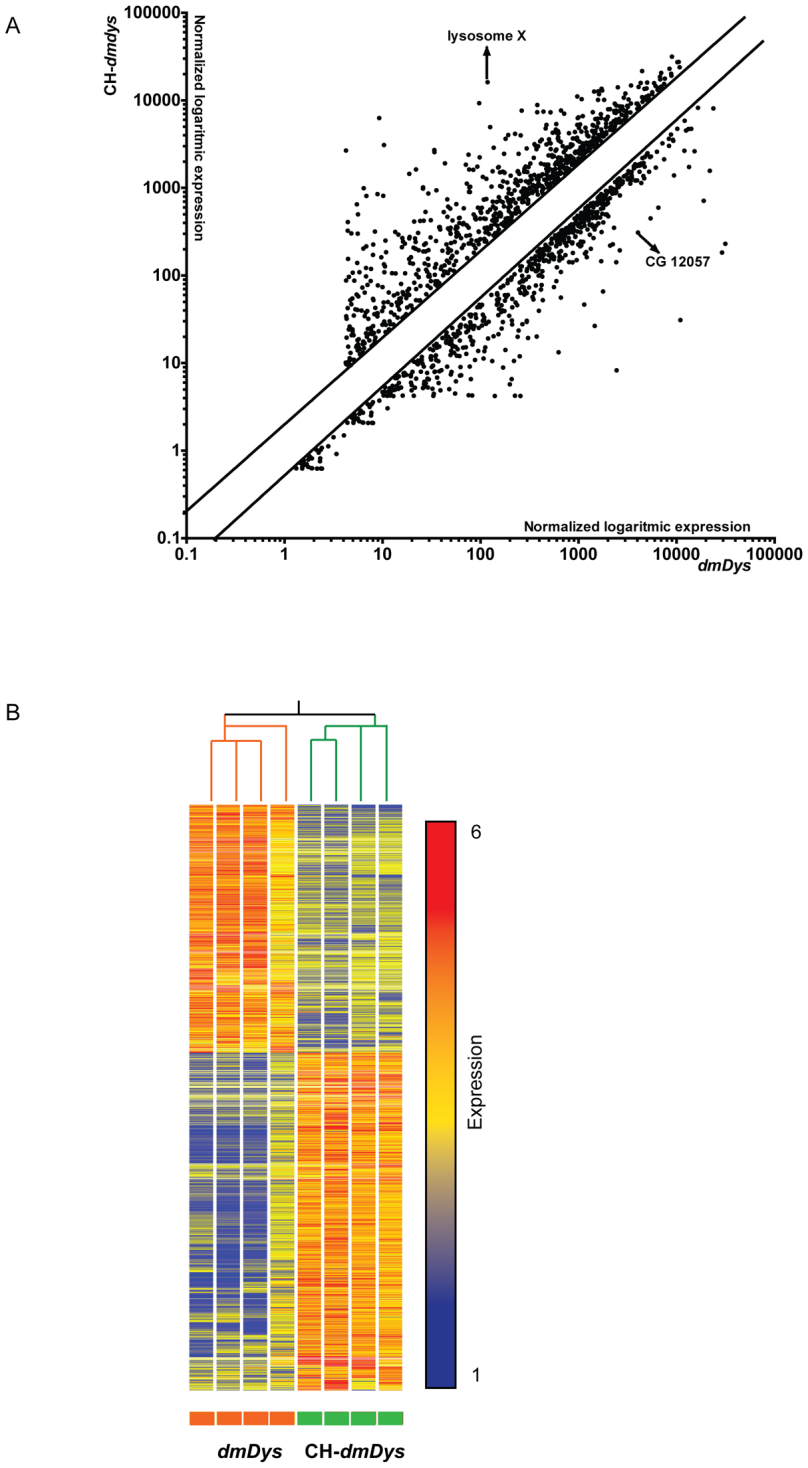
Differential expression of transcripts in *dmDys* exposed to chronic hypoxia. A) Scatter graph of log_10_ expression values of differentially expressed genes. Each individual point on the scatter graph represents a probe set that met the statistical and two-fold differential expression cut offs used in this study. Parallel lines show the 2 fold cutoff. Genes lying furthest off the diagonal exhibit greatest expression differences between CH-dm*Dys* and normoxic *dmDys*. The arrows indicate representative differentially expressed genes used for validation. B) Graphical representation of all 1281 transcripts that were differentially expressed in *CH-dmDys* and normoxic *dmDys*. The four *CH-dmDys* and four *dmDys* GeneChip data sets can be seen to cluster into two distinct groups based on correlation of gene expression pattern. The branches lengths for *CH-dmDys* and *dmDys* subtrees seen at the top are based on normalized raw data of all transcripts and quantitatively demonstrate that the four samples are closely related to each other, as are the four *dmDys* samples. Each horizontal colored bar represents one probe set, and the color of the bar determines the degree of expression (red  =  up-regulated genes; blue  =  down-regulated genes; yellow  =  no differentially regulated genes).

**Figure 2:**
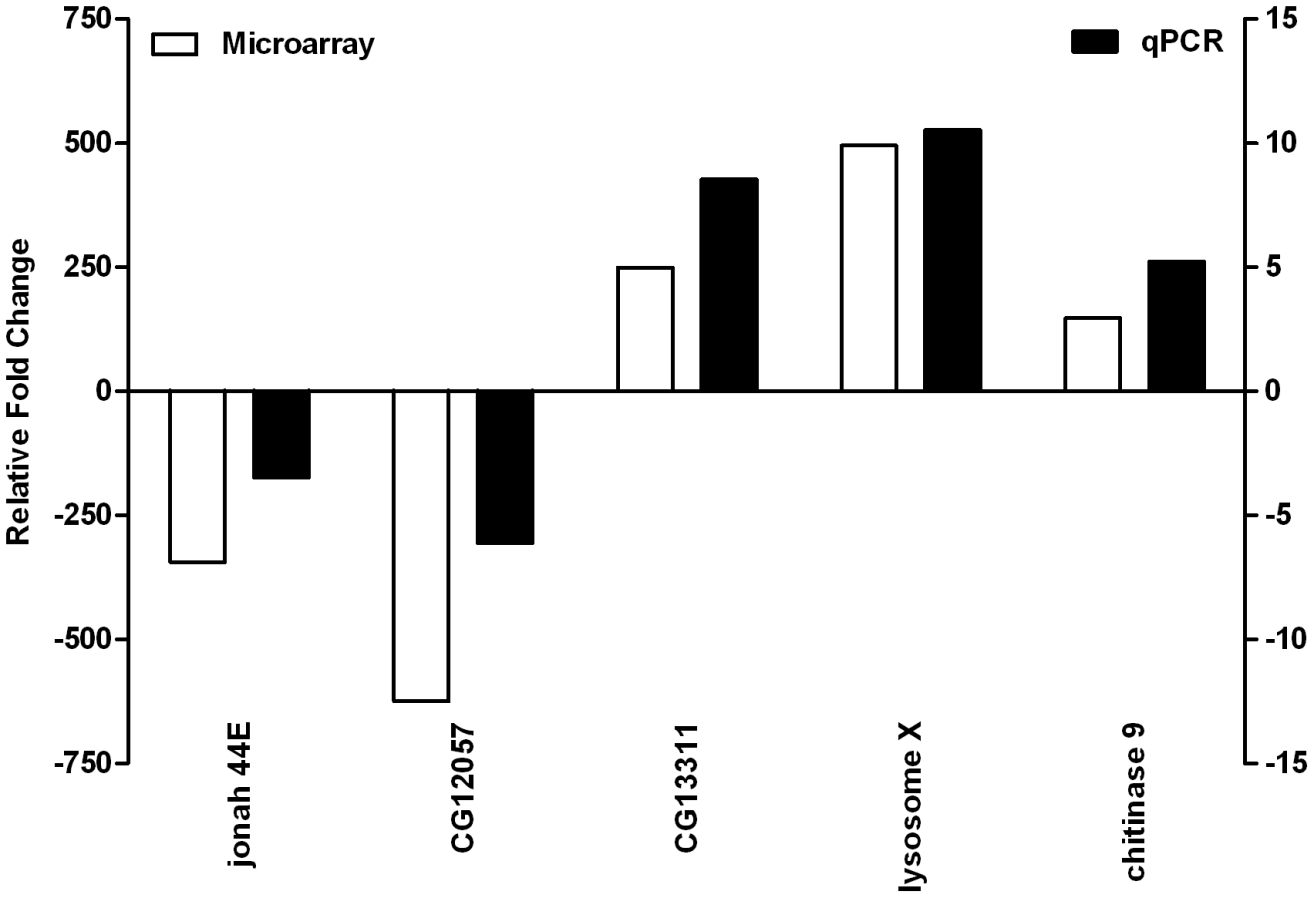
Validation of differential expression of five genes detected on *CH-dmDys* profile by real time RT-PCR. Five out of top 10 differentially expressed genes were amplified using cDNA from four independent RNA preparations and analyzed by qPCR. Graph shows concordant changes of gene expression levels for various genes noted on microarrays (unfilled bars) and validated by qPCR (filled bars).

To determine the effect of CH on dystrophin-deficient flies we screened the Affymetrix Drosophila Genome 2.0 GeneChip® platform and identified gene expression profiles of four individual CH-*dmDys* (Hypoxia protocol provided in Table S1) with four individual normoxic *dmDys* flies. The expression levels of all probe sets represented on individual microarrays was plotted on a scatter graph (Figure 1A) and shows the overall pattern of differences in gene expression. The overall correlation coefficient (r^2^) for the normoxic sample data sets compared amongst themselves was 0.98, for the hypoxic sets 0.97 and the r^2^ between the normoxic and hypoxic sample data sets was 0.86, demonstrating the low intra-variability among the samples from normoxic and hypoxic transcriptomes in the *dmDys* flies. After imposing statistical and 2 fold expression levels cutoffs, 1281 genes were found to be differentially expressed (Figure 1A and Table S3), of which 706 genes were significantly up-regulated (55.1% of the differentially expressed genes) and 575 significantly down-regulated (44.9% of the total differentially expressed). The genes for lysosome X and CG12057 were the most up-regulated (498.87 fold) and down-regulated (-624.6 fold), respectively. Figure 1B shows the heat map representation of hierarchical clustering of the profile. Branch-length analysis of data (Figure 1B) demonstrated that the four CH-*dmDys* samples were more similar to each other than to normoxic-*dmDys* samples and vice versa, further demonstrating that the gene expression profiles between CH- and normoxic-dmDys were significantly different from each other. Similar results were obtained using principal component analysis (data not shown). To independently validate the microarray analysis, we randomly chose five out of the top 10 differentially expressed genes (Table S4) and analyzed them by qPCR from biologically independent samples using TaqMan® Gene Expression probe sets. All five genes showed significant fold-changes in a direction concordant to that as observed in the microarray, confirming the validity of the results (Figure 2).

In order to understand the relationship between the genes differentially expressed and pathways involved we analyzed the differentially expressed genes list by clustering of functional annotation of the main biological processes using DAVID. To increase stringency, we only selected the enriched groups in which all annotations were significantly clustered (p<0.05). The most enriched categories among the up-regulated genes were amino acid, carboxylic acid and organic acid metabolic processes and among the down-regulated genes were regulation of protein kinase, transferase and kinase activities (Table S5).

**Figure 3:**
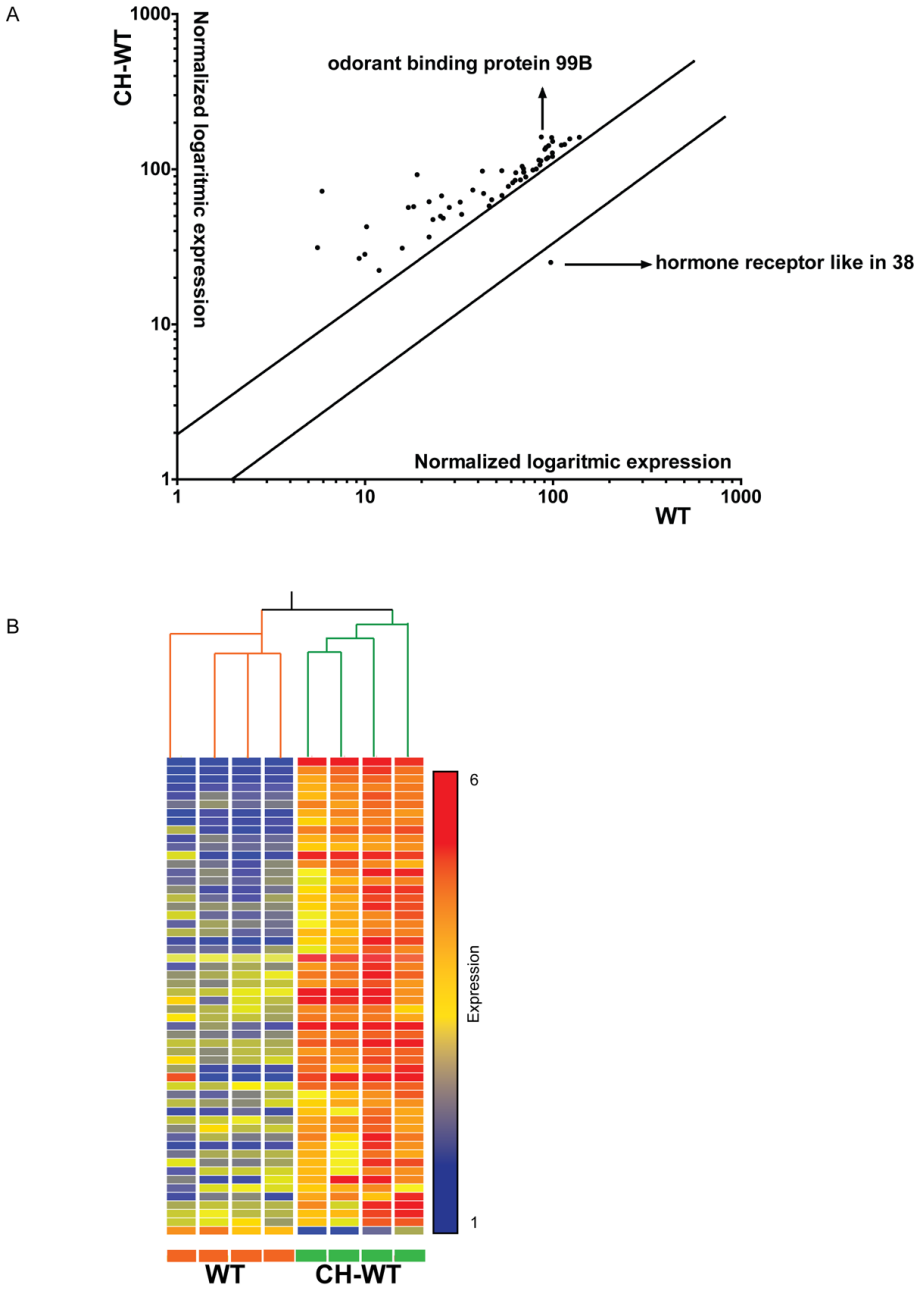
Differential expression of transcripts in WT exposed to chronic hypoxia. A) Scatter graph log_10_ expression values of differentially expressed genes. Each individual point on the scatter graph represents a probe set that met the statistical and two-fold differential expression cut offs used in this study. Parallel lines show the 2 fold cutoff. Genes lying furthest off the diagonal exhibit greatest expression differences between CH-WT and normoxic WT. The arrows indicate representative differentially expressed genes used for validation. B) Graphical representation of all 56 transcripts that were differentially expressed in CH-WT and normoxic WT. The four CH-WT and four WT GeneChip data sets can be seen to cluster into two distinct groups based on correlation of gene expression pattern. The branches lengths for CH-WT and WT subtrees seen at the top are based on normalized raw data of all transcripts and quantitatively demonstrate that the four samples are closely related to each other, as are the four WT samples. Each horizontal colored bar represents one probe set, and the color of the bar determines the degree of expression (red  =  up-regulated genes; blue  =  down-regulated genes; yellow  =  no differentially regulated genes).

**Figure 4:**
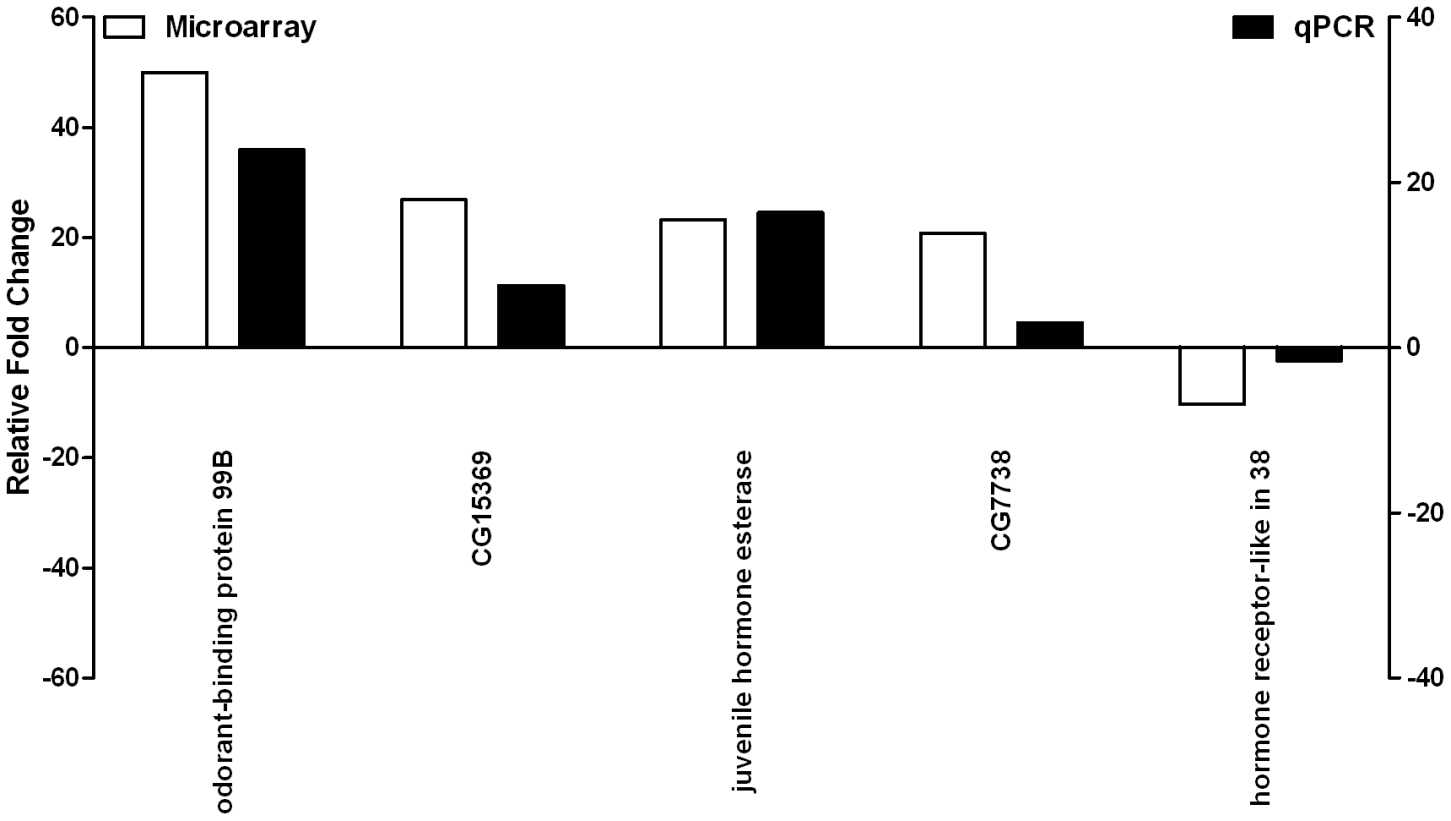
Validation of differential expression of five genes detected on WT profile by real time RT-PCR. Five out of top 10 differentially expressed genes were amplified using cDNA from four independent RNA preparations and analyzed by qPCR. Graph shows concordant changes of gene expression levels for various genes noted on microarrays (unfilled bars) and validated by qPCR (filled bars).

Having established the gene expression profile of *dmDys* subjected to CH we also determined the gene expression profile of CH wild type (WT) *Drosophila* in order to compare hypoxia-induced gene changes in *dmDys* to those in WT. We screened the Affymetrix Drosophila Genome 2.0 GeneChip® platform and identified gene expression profiles of four individual CH-WT (Hypoxia protocol provided in Table 1) flies with four individual normoxic WT flies. The expression levels of all probe sets represented on individual microarrays was plotted on a scatter graph (Figure 3A) and shows the overall pattern of differences in gene expression. The overall r^2^ for the normoxic sample data sets compared amongst themselves was 0.98, for the hypoxic sets 0.93 and the r^2^ between the normoxic and hypoxic sample data sets was 0.93, demonstrating the low intra-variability among the samples from normoxic and hypoxic transcriptomes in the WT flies. After imposing statistical and 2 fold expression levels cutoffs, 56 genes were found to be differentially expressed (Figure 3A; Table S6); 55 were up-regulated and only one gene was down-regulated. Figure 3B shows the heat map representation of hierarchical clustering of the entire profile. Branch-length analysis of data (Figure 3B) demonstrated that the four CH-WT samples were more similar to each other than to normoxic-WT samples, and vice versa demonstrating that the gene expression profiles between CH- and normoxic-WT were significantly different from each other. Similar results were obtained using principal component analysis (data not shown). To independently validate the microarray analysis randomly chose five (4 up-regulated and 1 down-regulated) out of the top 10 differentially expressed genes (Table S7) and analyzed them by qPCR from biologically independent samples using TaqMan® Gene Expression probe sets. All five genes showed significant fold-changes in a direction concordant to that as observed in the microarray, confirming the validity of the results (Figure 4). Functional clustering analysis using DAVID was performed only on the up-regulated gene list, and show an enrichment of the category related with response to stress (Table S8).

**Table 1:** Intersection gene list comparing profiles of WT and *dmDys* flies exposed to CH.

AFFY_ID	Gene Name	wild type	*dmDys*
**AFFX-r2-Dros-Act5C-5_x_at**	ACTIN 5C	4.11	124.77
**1641681_s_at**	CG5288-PA, isoform A	2.50	3.62
**1633637_at**	CG17026-PA	4.01	6.16
**1633197_at**	CG32024-PA	2.90	41.94
**1628699_at**	larval serum protein 1	6.36	4.76
**1637833_at**	CG30095-PA	23.22	7.47
**1637833_at**	juvenile hormone esterase	3.57	8.21
**1623675_at**	odorant-binding protein 99B	23.21	27.77
**1634529_at**	CG3290-PA	6.11	21
**1636620_s_at**	CG15095-PB, isoform B	2.86	2.65
**1638484_at**	heat shock protein 67BC	5.98	−14.63
**1635044_at**	heat shock protein 26	5.47	−12.12
**1638872_at**	heat shock protein 68	4.82	−24.55
**1629061_s_at**	heat shock protein 22	4.94	−9.98
**1641055_at**	heat shock protein 23	3.24	−7.31
**1628117_at**	heat shock protein 27	3.03	−5.63
**1634187_x_at**	alpha gamma-element	4.63	−18.95
**1626821_s_at**	heat shock protein 70BA	11.40	−2631.58
**1633770_at**	GH10821P	4.61	−3.54
**1639571_s_at**	heat shock protein 70AB	7.34	−2173.91

The AFFY ID, gene name and fold change values obtained from microarray analysis.

**Figure 5:**
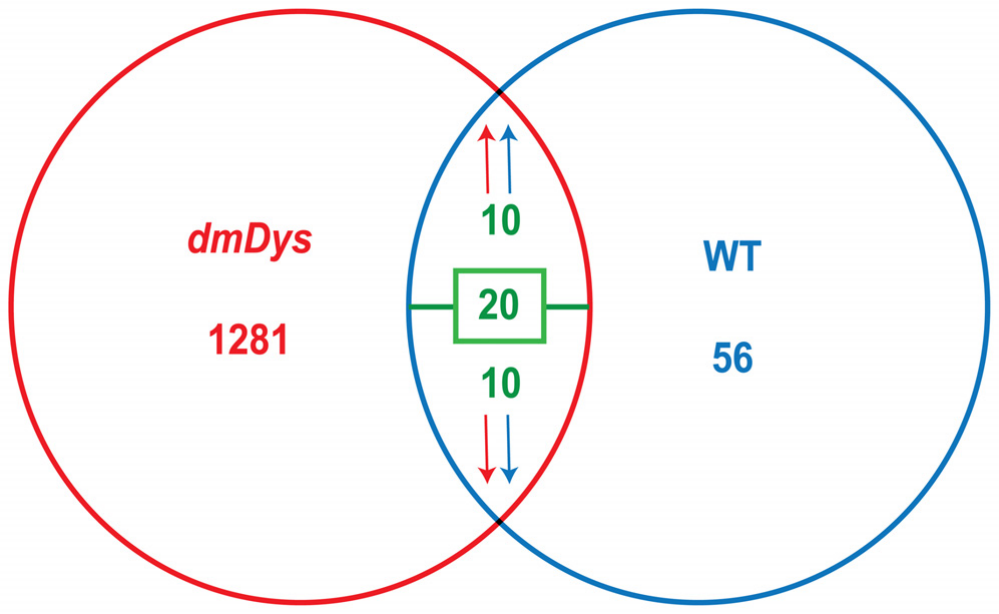
Venn diagram of comparison gene expression profile between *dmDys* and WT flies exposed to CH. From 1281 differentially expressed genes in dmDys and 56 in WT flies, 20 genes were found to be in common; among them, 10 genes were concordantly regulated in both profiles, while 10 were discordantly regulated in the profiles.

Comparing hypoxia-induced gene changes in *dmDys* and WT flies, we found 20 common genes (Figure 5), of which 10 genes were up-regulated in both profiles (CH-*dmDys* and CH-WT), and 10 genes were discordantly regulated in that they were down-regulated in CH-*dmDys* but up-regulated in CH-WT. Interestingly, 8 out of 10 genes in the disparate group belong to HSP family (Table 1). To determine the changes in gene expression caused only by the absence of dystrophin, we compare the gene expression profiles obtained from *dmDys vs.* WT, both under normoxic conditions. In this analysis, we found 208 genes differentially expressed, in which 114 genes were up-regulated and 94 were down-regulated in *dmDys* (Table S9). Interestedly, among the 114 up-regulated genes we found 8 Hsp genes, which were the same as those that were down-regulated in *dmDys* exposed to CH, as described above (Hsp23, Hsp26, Hsp27, Hsp67Bb, Hsp68, Hsp70Aa, Hsp70Bbb and Hsp70Bc).

The gene expression of two categories were analyzed in order to ask whether hypoxia was the major abiotic variable between the hypobaric hypoxic (Mount Denali) experiments and normobaric hypoxic experiments under controlled abiotic parameters. As shown on Table 2, the genes related to response to stress (Hsp23, Hsp26, Hsp27, Hsp67, Hsp67BC and Hsp68) showed an overall concordant down-regulation of expression in *dmDys* flies under both, normobaric or hypobaric hypoxic conditions. Moreover, the genes related to carbohydrate metabolism (Hexokinase C, Mannosidase 1, CG5288 and CG17026-PA) were consistently up-regulated in *dmDys* exposed to normobaric or hypobaric hypoxia. We also studied the gene expression level of two genes that were also present in both hypobaric and normobaric gene expression profiling. Odorant binding protein 99B (developmental process) and Juvenile hormone esterase (lipid metabolism) also showed a similar tendency of expression in both types of hypoxia. These results suggest that long term exposure of hypoxia was the major abiotic variable in the experimental paradigms.

**Table 2:** Validation of changes in expression levels of genes encoding heat shock proteins carbohydrate metabolism, developmental process and lipid metabolism in *dmDys* exposed to hypobaric and normobaric CH.

Category	Gene	CH-*dmDys*-Hypobaric	CH-*dmDys*-Normobaric
Response to stress	Hsp 23	−7.34±0.04*	−1.23±0.16
	Hsp 26	−7.74±0.023**	−2.45±0.28*
	Hsp 27	−6.24±0.05***	−2.66±0.11***
	Hsp67	−624.26±0.01***	−19.55±0.02**
	Hsp67BC	−64.84±0.01 ***	−14.41±0.01***
	Hsp68	−89.08±0.03***	−6.99±0.05*
Carbohydrate Metabolism	Hexokinase C	4.64±1.12*	4.74±0.70***
	Mannosidase 1	3.84±1.01*	4.16±1.45*
	CG5288	5.42±1.48*	2.25±0.53 **
	CG17026-PA	6.16±2.56 *	6.00±2.28*
Developmental process	Odorant binding protein 99B	8.57±3.24 **	1.84±0.63*
Lipid metabolism	Juvenile hormone esterase	1.37±0.31	1.18±0.41

Concordant decreases in levels of genes encoding HSP expression and increases in genes encoding carbohydrate metabolism, developmental process and lipid metabolism were noted in *dmDys* exposed to both hypobaric or normobaric hypoxia compared to normoxic *dmDys*. Values are mean ± SD.

*p<0.05.

**p<0.01;

***p<0.001 *vs.* normoxic *dmDys*.

### Functional Assays

#### Hypoxia-recovery assay

**Figure 6:**
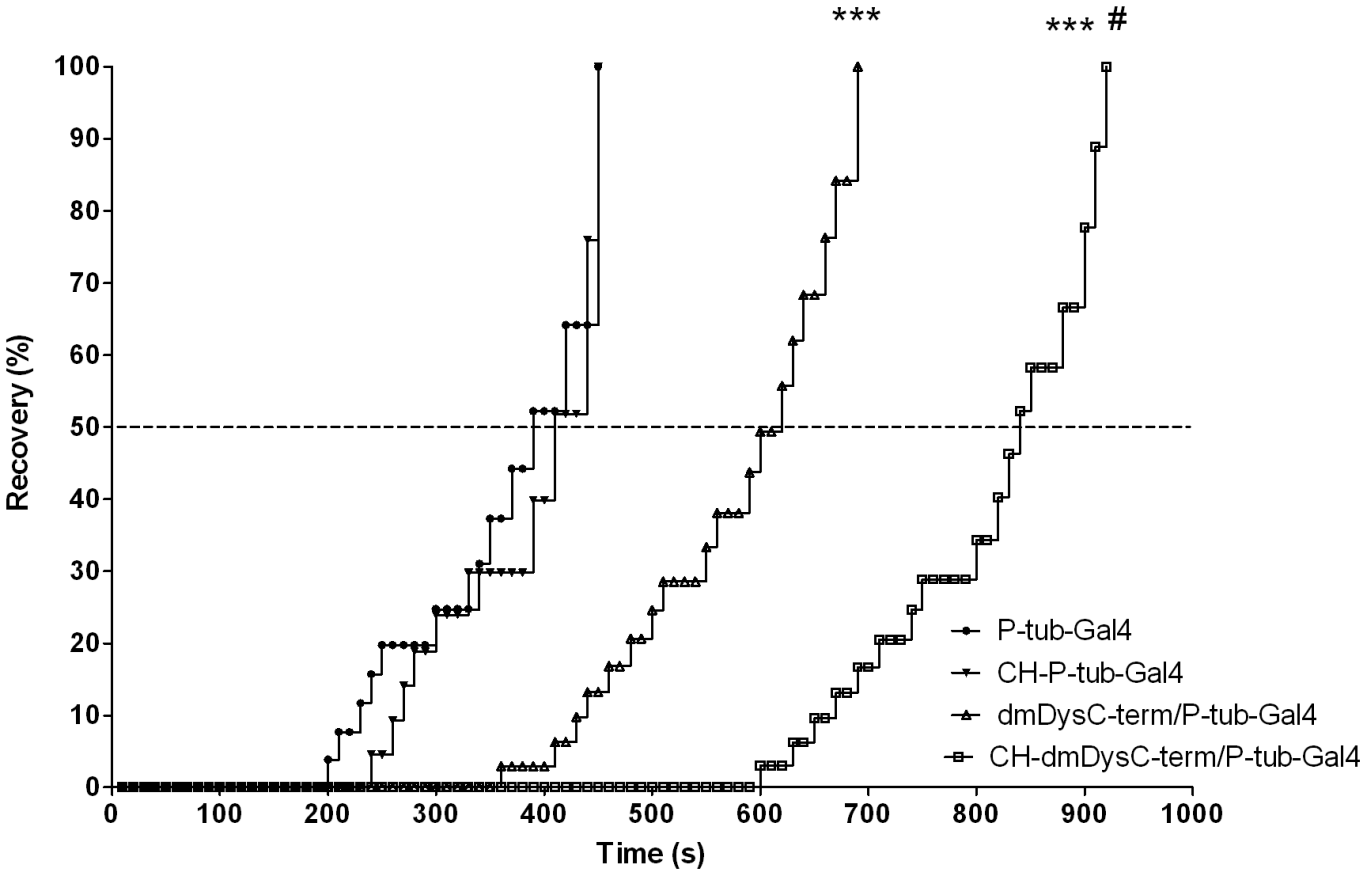
Time of recovery from severe hypoxic challenge assay. The *dmDysC-term* flies were exposed either to normoxia (triangle) or CH (square) following the hypoxia protocol. Then, the flies were exposed for 2 hours under 1% FiO_2_ and then to room air. The starting time was considered as the moment when the normoxia was reestablished and a complete recovery was considered when the fly climbed the vial. The driver *P-tub-Gal4* was used as control. Five vials from each genotype were used containing 20 flies per vial. The dotted line shows the median recovery time in seconds of the assay. *** p<0.001 *dmDys vs*. WT. # p<0.05 from *CH-dmDys vs.* normoxic *dmDys*.

In order to study functional effects of hypoxia in the absence of dystrophin, and address the possibility that the disparate molecular responses of dystrophin-deficient tissues to CH could adversely affect muscle function, we performed hypoxia-recovery and climbing assays. In the hypoxia-recovery assay we quantified the recovery time in seconds of controls, *dmDys* and *CH-dmDys* flies to determine whether a) dystrophin plays a role in recovery after being challenged by acute hypoxia and b) prior exposure to CH is detrimental to recovery from an acute hypoxic challenge. The WT (controls) had a faster median recovery time (c. 400 sec) in comparison to a slower median time to recovery (c.600 sec) of the *dmDysC-term/P-tub-Gal4* flies. This result showed a significantly impaired recovery by *dmDysC-term/P-tub-Gal4* flies after being challenged by acute severe hypoxia exposure (Figure 6). The median time to recover from the acute severe hypoxia was further increased in CH-*dmDys* (Table 3), demonstrating that prior exposure to CH is detrimental to recovery from an acute hypoxic challenge. This result shows the role played by dystrophin in *Drosophila* to normal behavioral recovery from hypoxia. Similar results were observed using muscle-specific driver (Figure S1-A), demonstrating that dystrophin expressed in muscle contributes to functional recovery after an acute hypoxic challenge. To obviate the possibility of alternate promoter usage we repeated the assays with both the transgenic drivers using N-terminal mutants of dystrophin and obtained similar results (Table 3; Figure S1-B and S1-C).

**Table 3:** Hypoxia recovery challenge assay in WT and *dmDys* flies previously exposed to normoxia or CH.

GENOTYPE	TIME (s)	P-VALUE
***P-tub-Gal4***	390	
***CH- P-tub-Gal4***	410	ns
***24B-Gal4***	400	
***CH-24B-Gal4***	410	ns
***dmDysC-term/P-tub-Gal4***	620	***
***CH- dmDysC-term/P-tubGal4***	840	***
***dmDysC-term/24B-Gal4***	600	***
***CH- dmDysC-term/24B-Gal4***	770	*** #
***dmDysN-term/P-tub-Gal4***	520	*** #
***CH-dmDysN-term/P-tub-Gal4***	660	***
***dmDysN-term/24B-Gal4***	590	***
***CH- dmDysN-term/24B-Gal4***	750	***

After acute hypoxic exposure (FiO_2_ 1% for 2 h), the time was recorded when the flies returned to normoxia until they climbed the vial. Median, n = 100.

***p<0.001 *dmDys vs*. WT.

# p<0.05 *dmDys vs.* CH-*dmDys*.

#### Climbing assay

We further analyzed the functional effects of hypoxia by subjecting the flies to climbing assays to test mobility of *dmDys*, as described previously [40]. In these assays, we quantified the climbing index of WT *vs. dmDys* and *vs.* CH-*dmDys* as an *in vivo* indirect measurement of innate muscle function to determine whether a) dystrophin plays a role in climbing mobility and b) prior exposure to CH is detrimental to mobility to climb in flies lacking dystrophin. As shown in Table 4, WT controls had a climbing index of c. 49%. In comparison to the controls, the *dmDysC-term/P-tub-Gal4* flies lacking dystrophin had a significantly impaired climbing index (c.11%), demonstrating the role played by dystrophin in terms of climbing ability (Figure S2-A). The climbing index even poorer in CH-*dmDys* (c. 8%), suggesting that prior exposure to CH is detrimental to innate muscle function as measured by the climbing index. These experiments were also performed using the strains and drivers described above and provided results similar to those obtained using the hypoxia recovery assay for *dmDysC-term* (Figure S2-B) and *dmDysN-term* (Figure S2-C and S2-D) genotypes (Table 4). Taken together these data demonstrate that dystrophin plays an important functional role in muscle and that CH further exacerbates the impaired function in the absence of dystrophin.

**Table 4:** Climbing assay in *dmDysC-term* exposed either to normoxia or CH.

GENOTYPE	CLIMBING INDEX (%)	P-VALUE
***P-tub-Gal4***	49.81±7.20	
***CH- P-tub-Gal4***	38.77±1.60	ns
***24B-Gal4***	30.36±9.38	
***CH-24B-Gal4***	38.74±9.45	ns
***dmDysC-term/P-tub-Gal4***	11.78±5.37	***
***CH-dmDysC-term/P-tubGal4***	8.02±3.65	**
***dmDysC-term/24B-Gal4***	3.70±2.14	*
***CH-dmDysC-term/24B-Gal4***	2.00±1.35	**
***dmDysN-term/P-tub-Gal4***	37.79±5.27	**
***CH-dmDysN-term/P-tub-Gal4***	15.57±2.37	**
***dmDysN-term/24B-Gal4***	12.50±7.98	ns
***CH-dmDysN-term/24B-Gal4***	5.78±2.69	*

Five vials from each genotype containing 20 flies in each vial were used. The results were compared to WT. Mean ± SEM, N = 100;

*P<0.05;

**P<0.01;

***P<0.001 *dmDys* vs. driver.

## Discussion

In this study we investigated the effects of CH upon gene expression at the level of the transcriptome and functional activity of *Drosophila melanogaster* model for DMD. Using Affymetrix GeneChip®-based expression flies were able to identify and analyze molecular responses to hypoxia at the level of the transcriptome in *dmDys* and WT flies (Figure 1 and 3 Table S3, S4, S6 and S7), as well as independently validate transcriptome level changes by qPCR (Figure 2 and 4, & Table 1 and 2). The molecular responses to CH in WT and *dmDys* flies were found to be disparate in terms of the ability of the respond to the stresses of hypoxia (Figure 5). In this experiments related to gene expression profiling on *dmDys* exposed to hypobaric hypoxia (Mount McKinley/Denali), we do not discount the possibility that several abiotic factors (such as temperature, humidity, barometric pressure, etc.) could have some influence in the profile. Nevertheless, we observed that the major changes in the gene expression profiling were also observed in controlled situations (normobaric hypoxia, Table 2), suggesting the major abiotic factor is related to hypoxia. Using functional assays we found that dystrophin plays an important functional role in muscle and that CH further exacerbates the impaired function in the absence of dystrophin (Figure 6 and Tables 3 & 4). Taken together our data demonstrate that dystrophin–deficient flies mount a disparate, pathological response to hypoxia and that chronic exposure to hypoxia impairs muscle function dystrophin-deficient flies *in vivo*.

*Drosophila melanogaster* models are being increasingly used to better understand the patho-physiology of human diseases. Over the last decade, this model has been used to study the mechanisms of hypoxia and its physiological effects, which has given enormous insight into angiogenesis, tumor formation, metabolism, and stress mechanisms [57], [58], [59], [60], [61]. More recently, *Drosophila* has been used to understand the molecular and cellular pathogenesis of DMD [38], [40], [41], [42]. Despite hypoxia and respiratory failure being important features of DMD disease, a number of open questions exist regarding the patho-physiological responses to hypoxia in muscle functioning and dystrophin-deficiency *in vivo*. Recently, several groups have been studying the effect of the lack of dystrophin in *Drosophila melanogaster*. These studies have increased our understanding of the cellular role of dystrophin and its complex in the muscle and other tissues. Additionally, identify the consequence of dystrophin deficiency on the function of the central nervous system, photo-receptor path-finding, neuromuscular junction and muscle integrity [40], [41], [42], [62]. The availability of the Drosophila model of DMD also allowed us to address the effect of CH on global gene expression and muscle function in the *Drosophila* DMD model.

It is currently well established that CH leads to robust changes in overall gene expression in Drosophila, albeit using different time scales, oxygen levels and ages of flies compared to our studies [48], [63], [64]. Using adult WT flies, Liu et al (2006) also showed that different intensities of hypoxia or lengths of exposure can induce different patterns of gene expression. For extreme hypoxia at 0.5% O_2_ for 1 hour, 20 genes were differentially expressed, increasing to 79 genes when the time was increased up to 6 hours. The number of differentially expressed genes decreased to 47 genes after the adult WT flies were exposed for 6 hours to lesser (5% O_2_) degrees of hypoxia [48]. These data are consistent with our results where we found 56 genes differentially expressed in response to c. 2 weeks of 13% CH (Table S6). Interestingly *dmDys* flies had a greater magnitude of response in that 1281 genes were differentially expressed in response to CH (Table S3). Collectively, these data suggest that exposing *dmDys* to CH in contrast to exposing WT flies to CH induces a significant change on gene expression in order adapt the new cellular needs induced by stresses of flies lacking dystrophin. Supporting this notion the analysis of the most enriched clustering categories from CH-*dmDys* up-regulated genes list revealed an increase in transcript levels of genes related to protein metabolism, biosynthesis, and apoptosis. Additionally, the most enriched categories from the down-regulated gene list were response to stress, the cell cycle and regulation of protein kinase activity. Conversely, the response to stress category (containing the Hsp genes) in WT flies exposed to CH was the only enriched category identified from the up-regulated gene list (Table S8). Interestingly, only 20 differentially expressed genes were identified in common between CH-WT and CH-*dmDys* profiles, out of which, half were dysregulated, being down-regulated in *dmDys* flies exposed to CH.

Members of the Hsp gene family were prominent amongst the genes that were regulated in a disparate manner in *dmDys* exposed to CH (Figure 5). Comparing this study with previous reports [48], [65], we found that the HSP family gene was the most common differentially expressed genes in the WT profile (Hsp22, Hsp23, Hsp26, Hsp27 Hsp67Bc, Hsp68, Hsp70Ba and Hsp70AB), which were also down-regulated in the CH-*dmDys* gene expression profile and demonstrate the inability of the *dmDys* flies to mount an efficient response to the stress of hypoxia. The Hsp pathway is well studied in terms of response to stresses such as hypoxia. Typically cellular responses to hypoxic stress include an increase of Hsp RNA and protein expressions are mediated in part by the Heat Shock Factor (HSF) transcription factor [66], [67]. Under various stresses the inert HSP-HSF complex is thought to disassociate allowing HSF to up-regulate Hsp. Additionally, it has been shown that under hypoxic conditions, HSF is actively transcribed by HIF due to the presence of two HRE sequences on intronic region of HSF gene [65]. In mammals Hsps have been classified into five families according to their molecular weight (small HSP, HSP60, HSP70, HSP90 and HSP100). HSPs provide tolerance not only to hyperthermia, but also resistance to hypoxia, ischemia, inflammation, among other stress conditions. Moreover, as chaperones, HSPs have other functions such as aiding correct protein folding, regulating protein degradation and in translocation of proteins to different cellular compartments [68]. In our studies we detected genes from the major Hsp families: the small HSP (Hsp22, Hsp23, Hsp26 and Hsp27) and HSP70 (Hsp67BC, Hsp70AB, Hsp70BA and Hsp70BBB) families [69]. Recently, it has been shown that sustained hypoxia (FiO_2_  = 1.0% for 2 h) increases the expression of Hsp 70 family, Hsp68 and Hsp23. This increase was associated with a higher survival rate of *Drosophila* exposed to prolonged hypoxic paradigm (FiO_2_  = 1.5% for 7 days). Mutants that have no copies or few copies of mRNA of Hsp70 were unable to survive, while over expression of Hsp70 on specific parts of the brain (mushroom body and antennal lobes) or heart (cardial cells, pericardial cells and hemocytes) significantly increased survival in CH [70]. However, the mechanisms and signaling pathways for protection given by high levels of expression of HSP still remain unknown. Interestingly, increased expression of HSP90 has been described in DMD patients muscle samples compared to control [71]. Moreover, the increased expression of HSP90 has been observed in regenerating fibers while increased levels of HSP72, HSP73 and HSP60 has been noted in hypercontracted fibers [72]. However, a reduction in expression of HSP20, GRP75 (HSP-70 family) and HSP90 has been reported using proteomic analysis of the diaphragm of the *mdx* mouse model of DMD; the same analysis also revealed an increased expression of cvHSP and HSP110 [73]. In view of the disparate responses of HSP in the CH-*dmDys* flies found in this study, it may be beneficial to re-evaluating the issue of HSP in clinical biopsies with regards to presence or absence of hypoxemia.

When comparing our hypobaric hypoxia CH-*dmDys* gene expression profiling data to data sets of responses to hypoxic exposure of mammalian muscle (See Lundby *et al.*)[74], two genes were found in common. The PDGF-and-VEGF related factor 2 was down-regulated 10.66 times and Hexokinase C up-regulated 2.78 times. In the comparison between normoxic WT and CH WT flies, the insulin-like peptide 5 was up-regulated 6.59 times. Finally, in the gene list of normoxic dystrophic flies compared to normoxic WT, the carbonic anhydrase 2 was up-regulated 2.67 times was found. In terms of functional similarity of genes described in mammals with those found in our study in CH-dm*Dys*, we found significant enriched clusters of up-regulated genes related with amino acid metabolism, chitin-related metabolism, cell cycle process and among the most down-regulated genes, we found the response to stress. For WT flies, the most significant enriched cluster is related to response to stress. These comparisons showed that the CH protocol used in our study induces different clusters of genes and differential responses compared to CH protocols using mild but prolonged hypoxic challenge.

It is well known that hypoxia has a profound effect on muscle performance in mammals [75], [76], [77], [78], [79] and hypoxia is able to alter behavioral and muscle function in flies [40], [53], [63], [64]. Additionally, it has also been well documented that the absence of dystrophin causes both functional and behavioral impairments [40], [41], [42], [80]. In our studies, we found that CH impaired both the ability to recover from hypoxia and climbing mobility performance in WT and *dmDys* flies. Importantly the mobility in either recovery from acute hypoxia exposure or the mobility to climb in flies lacking dystrophin was more pronounced (Tables 3 and 4). Furthermore, *dmDys* flies where we had ablated dystrophin only in the muscle, performed extremely poorly compared to WT, suggesting that muscle was an important functional target of the CH. Taken together the results would suggest that dystrophin plays an important functional role in muscle and that CH exacerbates impaired function in the absence of muscle dystrophin. The presence of short dystrophin isoforms (DP186, DP205 and DP117) in *dmDysN-term* mutants would also be expected to partially help recovery of muscle function and protection from hypoxic damage by facilitating somatic muscle contraction and hence ventilation [50], [81], [82], once the *dmDys* flies were returned to normoxia. This notion is supported by previous studies that have showed age-dependent muscle degeneration, loss of fiber density and vacuolization in of the indirect muscle fly [40] and increases in areas devoid of myofibrils in *dmDys* mutants fly hearts [80].

In conclusion, here we identified the distinct gene expression profiles of WT and *dmDys* flies exposed to hypoxia. We demonstrated that CH induces a disparate molecular response in the absence of dystrophin *in vivo*. The HSP pathway was identified as dysregulated in the response mounted by flies lacking dystrophin to hypoxia. The lack of a robust HSP induction in response to hypoxia may contribute to the functional impairment noted in the flies lacking dystrophin. As the HSP response is evolutionarily conserved, we suggest that this pathway may be of consequence in advanced DMD patients with a severe degree of hypoxemia. We hypothesize that targeting and correcting genes involved in the disparate molecular response to hypoxia may be a novel therapeutic strategy in DMD.

## Supporting Information

Table S1 Expedition log book for Mount Denali/McKinley Hypoxia Research Expedition. Information obtained during the ascent and summit of Denali, June 1st to June 16th of 2007. The oxygen pressure (PO2) was calculated from the barometric pressure. Load Ferry (LF) refers to a climb with loads to the specified highpoint and return to the starting point. The time of exposure is indicated in brackets.
(0.02 MB PDF)

Table S2 Genes tested by qPCR for microarray validation. Name, catalog number from Applied Biosystems® and REFSEQ from each gene used on validation of microarray study. The last gene is the housekeeping gene used.
(0.01 MB PDF)

Table S3 DmDys gene list.
(0.16 MB XLS)

Table S4 List of top 10 differentially expressed genes detected in dmDys exposed to CH profiling. Affy ID, name, FlyBase ID and fold change is shown for each gene.
(0.02 MB PDF)

Table S5 Functional Annotation from dmDys exposed to CH.
(0.03 MB XLS)

Table S6 WT gene list.
(0.02 MB XLS)

Table S7 List of top 10 differentially expressed genes expression detected in WT flies exposed to CH profiling. Affy ID, name, FlyBase ID and fold change is shown for each gene.
(0.01 MB PDF)

Table S8 Functional Annotation from WT exposed to CH.
(0.02 MB XLS)

Table S9 Nomoxic dmDys vs. WT gene list.
(0.05 MB XLS)

Figure S1 Time of recovery from severe hypoxic challenge assay. The dmDysC-term and dmDysN-term driven by either P-tub-Gal4 or 24B-Gal4 were exposed either to normoxia (triangle) or CH (square) following the hypoxia protocol. Then, the flies were exposed for 2 hours under 1% FiO2 and then to room air. The starting time was considered as the moment when the normoxia was reestablished and a complete recovery was considered when the fly climbed the vial. The driver P-tub-Gal4 or 24B-Gal4 was used as control. Five vials from each genotype were used containing 20 flies per vial. The dotted line shows the median recovery time of the assay. *** p<0.001 dmDys vs. drivers. # p<0.05 from CH-dmDys vs. normoxic dmDys.
(0.05 MB PDF)

Figure S2 Climbing index of dmDys exposed to CH. The dmDysC-term and dmDysN-term driven by either P-tub-Gal4 or 24B-Gal4 were exposed either to normoxia (red) or CH (blue) following the hypoxia protocol. Five vials from each genotype containing 20 flies in each vial were used. DmDysC-term mutation was driven by driven by tubulin (A) or muscle-specific driver (B), and dmDysN-term driven by tubulin (D) or muscle-specific (E). The driver P-tub-Gal4 or 24B-Gal4 was used as control. The starting time was considered as the moment when the normoxia was reestablished and a complete recovery was considered when the fly climbed the vial. Mean ± SEM, n = 100; * p<0.05; ** p<0.01; *** p<0.001 dmDys vs. WT under the same condition.
(0.02 MB PDF)

## References

[1] Engel AG, Franzini-Armstrong C (1994). Myology..

[2] Hoffman EP, Brown RH, Kunkel LM (1987). Dystrophin: the protein product of the Duchene muscular dystrophy locus.. Cell.

[3] Koenig M, Monaco AP, Kunkel LM (1988). The complete sequence of dystrophin predicts a rod-shaped cytoskeletal protein.. Cell.

[4] Bach JR, Ishikawa Y, Kim H (1997). Prevention of pulmonary morbidity for patients with Duchenne muscular dystrophy.. Chest.

[5] Inkley SR, Oldenburg FC, Vignos PJ (1974). Pulmonary function in Duchenne muscular dystrophy related to stage of disease.. Am J Med.

[6] Mukoyama M, Kondo K, Hizawa K, Nishitani H (1987). Life spans of Duchenne muscular dystrophy patients in the hospital care program in Japan.. J Neurol Sci.

[7] Rideau Y, Gatin G, Bach J, Gines G (1983). Prolongation of life in Duchenne's muscular dystrophy.. Acta Neurol (Napoli).

[8] Beck J, Weinberg J, Hamnegård C-H, Spahija J, Olofson J (2006). Diaphragmatic function in advanced Duchenne muscular dystrophy.. Neuromuscular Disorders.

[9] De Bruin PF, Ueki J, Bush A, Khan Y, Watson A (1997). Diaphragm thickness and inspiratory strength in patients with Duchenne muscular dystrophy.. Thorax.

[10] Liu M, Mineo K, Hanayama K, Fujiwara T, Chino N (2003). Practical problems and management of seating through the clinical stages of Duchenne's muscular dystrophy.. Archives of Physical Medicine and Rehabilitation.

[11] De Bruin PF, Ueki J, Bush A, Y Manzur A, Watson A (2001). Inspiratory flow reserve in boys with Duchenne muscular dystrophy.. Pediatric Pulmonology.

[12] Phillips MF, Quinlivan RC, Edwards RH, Calverley PM (2001). Changes in spirometry over time as a prognostic marker in patients with Duchenne muscular dystrophy.. Am J Respir Crit Care Med.

[13] Chakkalakal JV, Thompson J, Parks RJ, Jasmin BJ (2005). Molecular, cellular, and pharmacological therapies for Duchenne/Becker muscular dystrophies.. FASEB J.

[14] Huang X, Poy F, Zhang R, Joachimiak A, Sudol M (2000). Structure of a WW domain containing fragment of dystrophin in complex with beta-dystroglycan.. Nat Struct Biol.

[15] Greener MJ, Roberts RG (2000). Conservation of components of the dystrophin complex in Drosophila.. FEBS Letters.

[16] Apel ED, Roberds SL, Campbell KP, Merlie JP (1995). Rapsyn may function as a link between the acetylcholine receptor and the agrin-binding dystrophin-associated glycoprotein complex.. Neuron.

[17] Yamada H, Chiba A, Endo T, Kobata A, Anderson LV (1996). Characterization of dystroglycan-laminin interaction in peripheral nerve.. Journal of Neurochemistry.

[18] Gee SH, Madhavan R, Levinson SR, Caldwell JH, Sealock R (1998). Interaction of muscle and brain sodium channels with multiple members of the syntrophin family of dystrophin-associated proteins.. J Neurosci.

[19] Schultz J, Hoffmuller U, Krause G, Ashurst J, Macias MJ (1998). Specific interactions between the syntrophin PDZ domain and voltage-gated sodium channels.. Nat Struct Biol.

[20] Bredt DS (1999). Endogenous nitric oxide synthesis: biological functions and pathophysiology.. Free Radic Res.

[21] Brenman JE, Chao DS, Gee SH, McGee AW, Craven SE (1996). Interaction of nitric oxide synthase with the postsynaptic density protein PSD-95 and alpha1-syntrophin mediated by PDZ domains.. Cell.

[22] Grady RM, Grange RW, Lau KS, Maimone MM, Nichol MC (1999). Role for [alpha]-dystrobrevin in the pathogenesis of dystrophin-dependent muscular dystrophies.. Nat Cell Biol.

[23] Haycock JW, MacNeil S, Jones P, Harris JB, Mantle D (1996). Oxidative damage to muscle protein in Duchenne muscular dystrophy.. Neuroreport.

[24] Sander M, Chavoshan B, Harris SA, Iannaccone ST, Stull JT (2000). Functional muscle ischemia in neuronal nitric oxide synthase-deficient skeletal muscle of children with Duchenne muscular dystrophy.

[25] Thomas GD, Sander M, Lau KS, Huang PL, Stull JT (1998). Impaired metabolic modulation of Î±-adrenergic vasoconstriction in dystrophin-deficient skeletal muscle.

[26] Mendell JR, Engel WK, Derrer EC (1971). Duchenne muscular dystrophy: functional ischemia reproduces its characteristic lesions.. Science.

[27] Parker JM, Mendell JR (1974). Proximal myopathy induced by 5-HT-imipramine simulates Duchenne dystrophy.. Nature.

[28] Barbe F, Quera-Salva MA, McCann C, Gajdos P, Raphael JC (1994). Sleep-related respiratory disturbances in patients with Duchenne muscular dystrophy.. Eur Respir J.

[29] Smith PE, Calverley PM, Edwards RH (1988). Hypoxemia during sleep in Duchenne muscular dystrophy.. Am Rev Respir Dis.

[30] Farkas GA, McCormick KM, G LE (2007). Episodic hypoxia exacerbates respiratory muscle dysfunction in DMD(mdx) mice.. Muscle & Nerve.

[31] Gosselin LE, Barkley JE, Spencer MJ, McCormick KM, Farkas GA (2003). Ventilatory dysfunction in mdx mice: impact of tumor necrosis factor-alpha deletion.. Muscle Nerve.

[32] Ishizaki M, Suga T, Kimura E, Shiota T, Kawano R (2008). Mdx respiratory impairment following fibrosis of the diaphragm.. Neuromuscular Disorders.

[33] Bulfield G, Siller WG, Wight PA, Moore KJ (1984). X chromosome-linked muscular dystrophy (mdx) in the mouse.. Proc Natl Acad Sci U S A.

[34] Ryder-Cook AS, Sicinski P, Thomas K, Davies KE, Worton RG, Barnard EA, Darlison MG, Barnard PJ (1988). Localization of the mdx mutation within the mouse dystrophin gene.. EMBO J.

[35] Cooper BJ, Winand NJ, Stedman H, Valentine BA, Hoffman EP (1988). The homologue of the Duchenne locus is defective in X-linked muscular dystrophy of dogs.. Nature.

[36] Bassett DI, Currie PD (2003). The zebrafish as a model for muscular dystrophy and congenital myopathy.. Hum Mol Genet.

[37] Bessou C, Giugia JB, Franks CJ, Holden-Dye L, Segalat L (1998). Mutations in the Caenorhabditis elegans dystrophin-like gene dys-1 lead to hyperactivity and suggest a link with cholinergic transmission.. Neurogenetics.

[38] Neuman S, Kaban A, Volk T, Yaffe D, Nudel U (2001). The dystrophin/utrophin homologues in Drosophila and in sea urchin.. Gene.

[39] Roberts RG, Bobrow M (1998). Dystrophins in vertebrates and invertebrates.. Hum Mol Genet.

[40] Shcherbata HR, Yatsenko AS, Patterson L, Sood VD, Nudel U (2007). Dissecting muscle and neuronal disorders in a Drosophila model of muscular dystrophy.. Embo J.

[41] van der Plas MC, Pilgram GS, de Jong AW, Bansraj MR, Fradkin LG (2007). Drosophila Dystrophin is required for integrity of the musculature.. Mech Dev.

[42] van der Plas MC, Pilgram GS, Plomp JJ, de Jong A, Fradkin LG (2006). Dystrophin is required for appropriate retrograde control of neurotransmitter release at the Drosophila neuromuscular junction.. J Neurosci.

[43] Jin H, Tan S, Hermanowski J, Bohm S, Pacheco S (2007). The dystrotelin, dystrophin and dystrobrevin superfamily: new paralogues and old isoforms.. BMC Genomics.

[44] Regulski M, Stasiv Y, Tully T, Enikolopov G (2004). Essential function of nitric oxide synthase in Drosophila.. Curr Biol.

[45] Nambu JR, Chen W, Hu S, Crews ST (1996). The Drosophila melanogaster similar bHLH-PAS gene encodes a protein related to human hypoxia-inducible factor 1 alpha and Drosophila single-minded.. Gene.

[46] Lavista-Llanos S, Centanin L, Irisarri M, Russo DM, Gleadle JM (2002). Control of the hypoxic response in Drosophila melanogaster by the basic helix-loop-helix PAS protein similar.. Mol Cell Biol.

[47] Sonnenfeld M, Ward M, Nystrom G, Mosher J, Stahl S (1997). The Drosophila tango gene encodes a bHLH-PAS protein that is orthologous to mammalian Arnt and controls CNS midline and tracheal development.. Development.

[48] Liu G, Roy J, Johnson EA (2006). Identification and function of hypoxia-response genes in Drosophila melanogaster.. Physiol Genomics.

[49] Gullan PJ, Cranston PS (1994). The insects: an outline of entomology..

[50] Consoulas C, Theophilidis G (1992). Anatomy, innervation and motor control of the abdominal dorsal muscles of Decticus albifrons (Orthoptera).. Journal of Insect Physiology.

[51] West JB (1996). Prediction of barometric pressures at high altitudes with the use of model atmospheres.. J Appl Physiol.

[52] Tusher VG, Tibshirani R, Chu G (2001). Significance analysis of microarrays applied to the ionizing radiation response.. Proc Natl Acad Sci U S A.

[53] Krishnan SN, Sun Y-A, Mohsenin A, Wyman RJ, Haddad GG (1997). Behavioral and Electrophysiologic Responses of Drosophila melanogaster to Prolonged Periods of Anoxia.. Journal of Insect Physiology.

[54] Benzer S (1967). Behavioral mutants of Drosophila isolated by countercurrent distribution.. Proc Natl Acad Sci U S A.

[55] Greene JC, Whitworth AJ, Kuo I, Andrews LA, Feany MB (2003). Mitochondrial pathology and apoptotic muscle degeneration in Drosophila parkin mutants.. Proc Natl Acad Sci U S A.

[56] Pfaffl MW, Horgan GW, Dempfle L (2002). Relative expression software tool (REST) for group-wise comparison and statistical analysis of relative expression results in real-time PCR.. Nucleic Acids Res.

[57] Abraham RT (2005). TOR Signaling: An Odyssey from Cellular Stress to the Cell Growth Machinery.. Current Biology.

[58] Horowitz A, Simons M (2008). Branching Morphogenesis.. Circ Res.

[59] Reddy BVVG, Irvine KD (2008). The Fat and Warts signaling pathways: new insights into their regulation, mechanism and conservation.. Development.

[60] Priti A, Gabriel GH (2009). Survival in Acute and Severe Low O2 Environment.. Annals of the New York Academy of Sciences.

[61] Scrable H, Medrano S, Ungewitter E (2009). Running on empty: How p53 controls INS/IGF signaling and affects life span.. Experimental Gerontology.

[62] Fradkin LG, Baines RA, van der Plas MC, Noordermeer JN (2008). The dystrophin Dp186 isoform regulates neurotransmitter release at a central synapse in Drosophila.. J Neurosci.

[63] Huang H, Haddad GG (2007). Drosophila dMRP4 regulates responsiveness to O2 deprivation and development under hypoxia.. Physiol Genomics.

[64] Zhou D, Xue J, Chen J, Morcillo P, Lambert JD (2007). Experimental selection for Drosophila survival in extremely low O2 environment.. PLoS ONE.

[65] Baird NA, Turnbull DW, Johnson EA (2006). Induction of the heat shock pathway during hypoxia requires regulation of heat shock factor by hypoxia-inducible factor-1.. J Biol Chem.

[66] Pelham HR (1982). A regulatory upstream promoter element in the Drosophila hsp 70 heat-shock gene.. Cell.

[67] Orosz A, Wisniewski J, Wu C (1996). Regulation of Drosophila heat shock factor trimerization: global sequence requirements and independence of nuclear localization.. Mol Cell Biol.

[68] Snoeckx LH, Cornelussen RN, Van Nieuwenhoven FA, Reneman RS, Van Der Vusse GJ (2001). Heat shock proteins and cardiovascular pathophysiology.. Physiol Rev.

[69] Michaud S, Marin R, Tanguay RM (1997). Regulation of heat shock gene induction and expression during Drosophila development.. Cellular and Molecular Life Sciences (CMLS).

[70] Azad P, Zhou D, Russo E, Haddad GG (2009). Distinct Mechanisms Underlying Tolerance to Intermittent and Constant Hypoxia in Drosophila melanogaster.. PLoS ONE.

[71] Bornman L, Polla BS, Gericke GS (1996). Heat-shock protein 90 and ubiquitin: developmental regulation during myogenesis.. Muscle Nerve.

[72] Bornman L, Polla BS, Lotz BP, Gericke GS (1995). Expression of heat-shock/stress proteins in Duchenne muscular dystrophy.. Muscle Nerve.

[73] Doran P, Martin G, Dowling P, Jockusch H, Ohlendieck K (2006). Proteome analysis of the dystrophin-deficient MDX diaphragm reveals a drastic increase in the heat shock protein cvHSP.. Proteomics.

[74] Lundby C, Calbet JA, Robach P (2009). The response of human skeletal muscle tissue to hypoxia.. Cell Mol Life Sci.

[75] West JB (2000). The physiologival challenge of climbing Mt. Everest.. Annals of the New York Academy of Sciences.

[76] West JB (2003). George I. Finch and his pioneering use of oxygen for climbing at extreme altitudes.. J Appl Physiol.

[77] Bailey DD, Bruce (1997). Physiological implications of altitude training for endurance performance at sea level: a review.. British Journal of Sport Medicine.

[78] Brutsaert T (2008). Do high-altitude natives have enhanced exercise performance at altitude?. Applied Physiology Nutritional and Metabolism.

[79] Askew EW (2002). Work at high altitude and oxidative stress: antioxidant nutrients.. Toxicology.

[80] Ouarda T-L, Takeshi A, Grant H, Uri N, David Y (2008). Dystrophin deficiency in Drosophila reduces lifespan and causes a dilated cardiomyopathy phenotype.. Aging Cell.

[81] Slama K (1988). A New Look at Insect Respiration.. Biol Bull.

[82] Bradley TJ (2008). Control of the Respiratory Pattern in Insects.. Hypoxia And The Circulation.

