# Supplementary material for: Chronic Hypoxia Impairs Muscle Function in the Drosophila Model of Duchenne's Muscular Dystrophy (DMD)
Source: PLoS One. 2010 Oct 20;5(10):e13450. doi: 10.1371/journal.pone.0013450 (PMC2958114; doi:10.1371/journal.pone.0013450)
Supplement: Table S1 — Expedition log book for Mount Denali/McKinley Hypoxia Research Expedition. Information obtained during the ascent and summit of Denali, June 1st to June 16th of 2007. The oxygen pressure (PO2) was calculated from the barometric pressure. Load Ferry (LF) refers to a climb with loads to the specified highpoint and return to the starting point. The time of exposure is indicated in brackets. (0.02 MB PDF) [file pone.0013450.s001.pdf]

Table S1. Expedition log book for Mount Denali / McKinley Hypoxia Research Expedition. Information obtained during the ascent and summit of Denali, June 1st to June 16th of 2007. The oxygen pressure (PO_2) was calculated from the barometric pressure. Load Ferry (LF) refers to a climb with loads to the specified highpoint and return to the starting point. The time of exposure is indicated in brackets.

DAY	LOCATION	ALTITUDE (m)	PO_2 mmHg (%)
1	Base Camp	2200	123.6 (16.3%)
2	Base Camp	2200	123.6 (16.3%)
3	Base Camp	2200	123.6 (16.3%)
4	Ski Hill	2400	120.7 (15.9%)
5	Kahiltna Pass	2950	113.0 (14.9%)
6	Motorcycle Hill	3350	107.7 (14.2%)
7	Motorcycle Hill	3350	107.7 (14.2%)
8	LF from Motorcycle	4150 (5 hours)	97.7 (12.9%)
9	Medical Camp	4350	95.3 (12.5%)
10	LF from Medical Camp	4150 (5 hours)	97.7 (12.9%)
11	Medical Camp	4350	95.3 (12.5%)
12	LF from Medical Camp	4900	89.0 (11.7%)
13	Medical Camp	4350	95.3 (12.5%)
14	High Camp	5250	85.1 (11.2%)
15	Summit	6194 (0.3 hours)	75.4 (9.9%)
16	High Camp	5250	85.1 (11.2%)
