# Supplementary material for: Chronic Hypoxia Impairs Muscle Function in the Drosophila Model of Duchenne's Muscular Dystrophy (DMD)
Source: PLoS One. 2010 Oct 20;5(10):e13450. doi: 10.1371/journal.pone.0013450 (PMC2958114; doi:10.1371/journal.pone.0013450)
Supplement: Table S2 — Genes tested by qPCR for microarray validation. Name, catalog number from Applied Biosystems® and REFSEQ from each gene used on validation of microarray study. The last gene is the housekeeping gene used. (0.01 MB PDF) [file pone.0013450.s002.pdf]

Table S2. List of genes used for microarray validation. Name, catalog number from Applied Biosystems® and REFSEQ from each gene used on validation of microarray study. The last gene is the housekeeping gene used.

NAME	TAQMAN GENE EXPRESSION ASSAY ID	REFSEQ
CG10531	Dm01827770_g1	NM_137699.3
CG13311	Dm02367367_s1	NM_140001.2
lysosome X	Dm01821925_s1	NM_079157.1
jonah 44E	Dm01812211_s1	NM_165618.1
CG12057	Dm01837328_s1	NM_132306.2
juvenile hormone esterase	Dm01821470_g1	NM_079034.2
CG15369	Dm018352_g1	NM_132314.1
odorant binding protein 99b	Dm02146202_s1	NM_143456.1
CG7738	Dm01819533_s1	NM_136809.3
hormone receptor-like in 38	Dm01842600_m1	NM_057771.2
ribosome protein like 11	Dm01842483-g1	NM_025919.5
