# Supplementary material for: Chronic Hypoxia Impairs Muscle Function in the Drosophila Model of Duchenne's Muscular Dystrophy (DMD)
Source: PLoS One. 2010 Oct 20;5(10):e13450. doi: 10.1371/journal.pone.0013450 (PMC2958114; doi:10.1371/journal.pone.0013450)
Supplement: Table S4 — List of top 10 differentially expressed genes detected in dmDys exposed to CH profiling. Affy ID, name, FlyBase ID and fold change is shown for each gene. (0.02 MB PDF) [file pone.0013450.s004.pdf]

Table S4. List of top 10 differentially expressed genes detected in *dmDys* exposed to CH profiling. Affy ID, name, FlyBase ID and fold change is shown for each gene.

Affy ID	NAME	FlyBase ID	FOLD CHANGE
1635270_at	CG14499-PA	FBgn0034317	708.34
1632720_at	lyzosome X	FBgn0004431	494.87
1634064_at	CG13311-PA	FBgn0035929	249.54
1630600_at	frost	FBgn0037724	168.94
1623027_s_at	CG6277-PA	FBgn0039475	159.84
1631446_at	chitinase 9	FBgn0034582	146.66
AFFX-r2-Dros-Act5C-5_at	actin 5C	FBgn0000042	124.77
1633749_at	CG6337	FBgn 0033873	124.15
1626386_at	cuticular protein 57A	FBgn0034517	115.12
1628541_at	tak-1 like 1	FBgn0046689	113.17
1624543_s_at	SD01615P	FBgn0029744	-253.68
1628617_at	drosomycin-4	FBgn0052282	-266.95
1637055_s_at	gtwin	FBte0001062	-297.44
1633617_at	CG15116-PA	FBgn0034415	-323.10
1639364_at	jonah 44E	FBgn0001285	-336.59
1634076_at	LP05863P	FBgn0030098	-624.61
1632841_x_at	heat shock protein 70Ba	FBgn001327	-836.12
1639571_s_at	heat shock protein 70BBB	FBgn0051354	-2132.20
1626821_s_at	heat shock protein 70AB	FBgn0013276	-2570.69

1635878_s_at	CG17571-PA.3	FBgn0032947	-2923.00
