# Supplementary material for: Chronic Hypoxia Impairs Muscle Function in the Drosophila Model of Duchenne's Muscular Dystrophy (DMD)
Source: PLoS One. 2010 Oct 20;5(10):e13450. doi: 10.1371/journal.pone.0013450 (PMC2958114; doi:10.1371/journal.pone.0013450)
Supplement: Table S7 — List of top 10 differentially expressed genes expression detected in WT flies exposed to CH profiling. Affy ID, name, FlyBase ID and fold change is shown for each gene. (0.01 MB PDF) [file pone.0013450.s007.pdf]

Table S7. List of top 10 differentially expressed genes expression detected in WT flies exposed to CH profiling. Affy ID, name, FlyBase ID and fold change is shown for each gene.

| Affy ID | NAME | FlyBase ID | FOLD CHANGE |
|---|---|---|---|
| 1623675_at | odorant-binding protein 99B | FBgn0039685 | 50.01 |
| 1639982_at | CG15369-PA | FBgn0030105 | 26.88 |
| 1637833_at | juvenile hormone esterase | FBgn0010052 | 23.22 |
| 1634525_at | CG7738-PA | FBgn0033596 | 20.77 |
| 1632841_x_at | heat shock protein 70 Ba | FBgn001327 | 11.83 |
| 1626821_s_at | heat shock protein 70 BBB | FBgn0051354 | 11.40 |
| 1626248_at | CG18547 | FBgn0037973 | 9.29 |
| 1630568_at | unknown | | 8.70 |
| 1629738_at | CG14954-RA | FBgn0052486 | 7.46 |
| 1639571_s_at | heat shock protein 70AB | FBgn0013276 | 7.34 |
| 163966_at | hormone receptor-like in 38 | FBgn0014859 | -10.31 |
