# Supplementary material for: Chronic Hypoxia Impairs Muscle Function in the Drosophila Model of Duchenne's Muscular Dystrophy (DMD)
Source: PLoS One. 2010 Oct 20;5(10):e13450. doi: 10.1371/journal.pone.0013450 (PMC2958114; doi:10.1371/journal.pone.0013450)
Supplement: Figure S1 — Time of recovery from severe hypoxic challenge assay. The dmDysC-term and dmDysN-term driven by either P-tub-Gal4 or 24B-Gal4 were exposed either to normoxia (triangle) or CH (square) following the hypoxia protocol. Then, the flies were exposed for 2 hours under 1% FiO2 and then to room air. The starting time was considered as the moment when the normoxia was reestablished and a complete recovery was considered when the fly climbed the vial. The driver P-tub-Gal4 or 24B-Gal4 was used as control. Five vials from each genotype were used containing 20 flies per vial. The dotted line shows the median recovery time of the assay. *** p<0.001 dmDys vs. drivers. # p<0.05 from CH-dmDys vs. normoxic dmDys. (0.05 MB PDF) [file pone.0013450.s010.pdf]

A

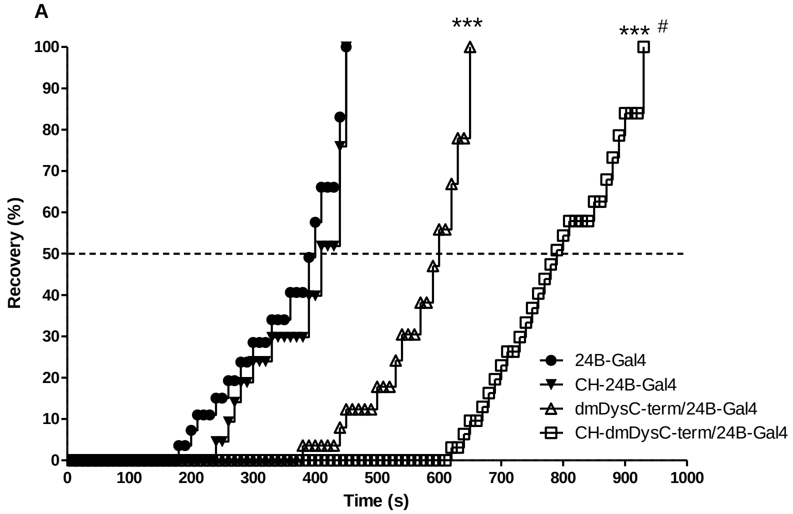
***
*** #
Recovery (%)
Time (s)
0
10
20
30
40
50
60
70
80
90
100
0
100
200
300
400
500
600
700
800
900
1000
24B-Gal4
CH-24B-Gal4
dmDysC-term/24B-Gal4
CH-dmDysC-term/24B-Gal4

B

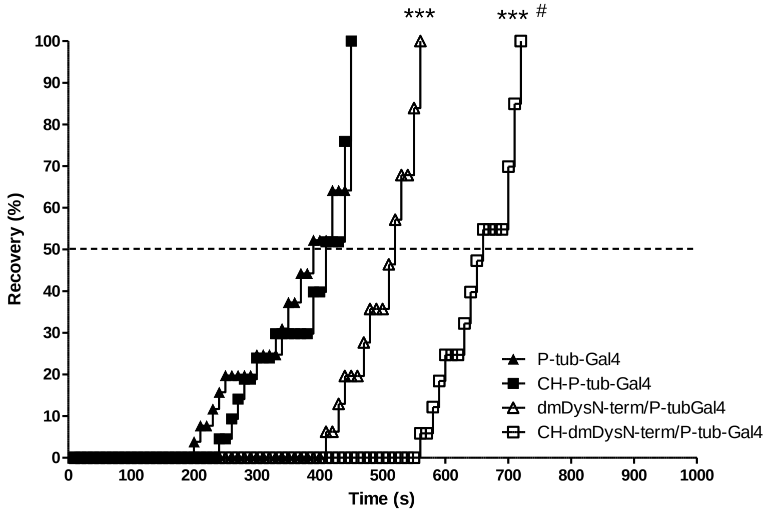
***
*** #
Recovery (%)
Time (s)
0
10
20
30
40
50
60
70
80
90
100
0
100
200
300
400
500
600
700
800
900
1000
P-tub-Gal4
CH-P-tub-Gal4
dmDysN-term/P-tubGal4
CH-dmDysN-term/P-tub-Gal4

C

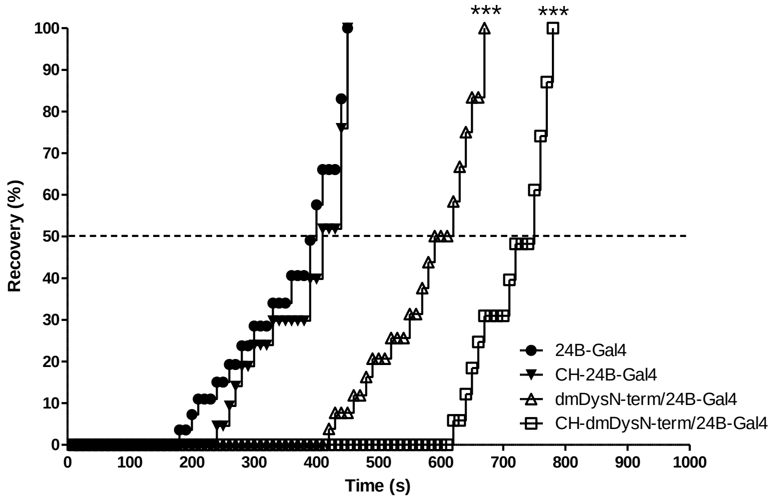
***
***
Recovery (%)
Time (s)
0
10
20
30
40
50
60
70
80
90
100
0
100
200
300
400
500
600
700
800
900
1000
24B-Gal4
CH-24B-Gal4
dmDysN-term/24B-Gal4
CH-dmDysN-term/24B-Gal4

Figure S1.Time of recovery from severe hypoxic challenge assay. The *dmDysC-term* and *dmDysN-term* driven by either *P-tub-Gal4* or *24B-Gal4* were exposed either to normoxia (triangle) or CH (square) following the hypoxia protocol. Then, the flies were exposed for 2 hours under 1% FiO_2 and then to room air. The starting time was considered as the moment when the normoxia was reestablished and a complete recovery was considered when the fly climbed the vial. The driver *P-tub-Gal4* or *24B-Gal4* was used as control. Five vials from each genotype were used containing 20 flies per vial. The dotted line shows the median recovery time of the assay. *** $p < 0.001$ *dmDys vs*. drivers. # $p < 0.05$ from *CH-dmDys vs* normoxic *dmDys*.
