# Supplementary material for: Chronic Hypoxia Impairs Muscle Function in the Drosophila Model of Duchenne's Muscular Dystrophy (DMD)
Source: PLoS One. 2010 Oct 20;5(10):e13450. doi: 10.1371/journal.pone.0013450 (PMC2958114; doi:10.1371/journal.pone.0013450)
Supplement: Figure S2 — Climbing index of dmDys exposed to CH. The dmDysC-term and dmDysN-term driven by either P-tub-Gal4 or 24B-Gal4 were exposed either to normoxia (red) or CH (blue) following the hypoxia protocol. Five vials from each genotype containing 20 flies in each vial were used. DmDysC-term mutation was driven by driven by tubulin (A) or muscle-specific driver (B), and dmDysN-term driven by tubulin (D) or muscle-specific (E). The driver P-tub-Gal4 or 24B-Gal4 was used as control. The starting time was considered as the moment when the normoxia was reestablished and a complete recovery was considered when the fly climbed the vial. Mean ± SEM, n = 100; * p<0.05; ** p<0.01; *** p<0.001 dmDys vs. WT under the same condition. (0.02 MB PDF) [file pone.0013450.s011.pdf]

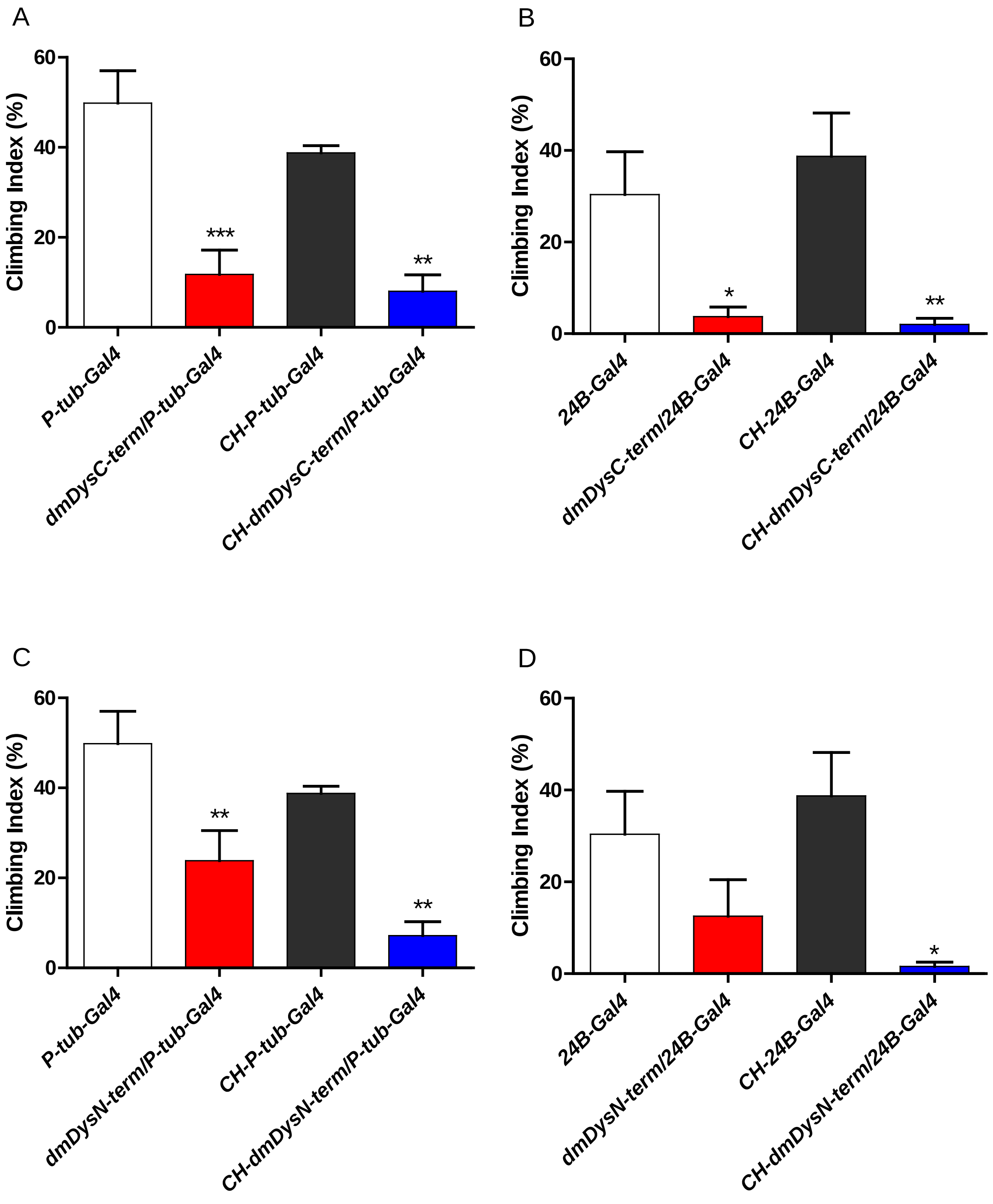

Figure S2. Climbing index of *dmDys* exposed to CH. The *dmDysC-term* and *dmDysN-term* driven by either *P-tub-Gal4* or *24B-Gal4* were exposed either to normoxia (red) or CH (blue) following the hypoxia protocol. Five vials from each genotype

containing 20 flies in each vial were used. *DmDysC-term* mutation was driven by driven by tubulin (A) or muscle-specific driver (B), and *dmDysN-term* driven by tubulin (D) or muscle-specific (E). The driver *P-tub-Gal4* or *24B-Gal4* was used as control. The starting time was considered as the moment when the normoxia was reestablished and a complete recovery was considered when the fly climbed the vial. Mean ± SEM, n = 100; * $p < 0.05$; ** $p < 0.01$; *** $p < 0.001$ *dmDys* vs. WT under the same condition.
